# Different Neutralization Profiles After Primary SARS-CoV-2 Omicron BA.1 and BA.2 Infections

**DOI:** 10.3389/fimmu.2022.946318

**Published:** 2022-07-19

**Authors:** Iris Medits, David N. Springer, Marianne Graninger, Jeremy V. Camp, Eva Höltl, Stephan W. Aberle, Marianna T. Traugott, Wolfgang Hoepler, Josef Deutsch, Oliver Lammel, Christian Borsodi, Elisabeth Puchhammer-Stöckl, Alexander Zoufaly, Lukas Weseslindtner, Judith H. Aberle, Karin Stiasny

**Affiliations:** ^1^ Center for Virology, Medical University of Vienna, Vienna, Austria; ^2^ Center for Public Health, Medical University of Vienna, Vienna, Austria; ^3^ Department of Medicine IV, Clinic Favoriten, Vienna Healthcare Group, Vienna, Austria; ^4^ Practice Dr. Deutsch, Völkermarkt, Austria; ^5^ Practice Dr. Lammel, Ramsau am Dachstein, Austria

**Keywords:** SARS-CoV-2, SARS-CoV-2 variant of concern, Omicron sub-lineages, SARS-CoV-2 neutralization, immune escape

## Abstract

**Background and Methods:**

The SARS-CoV-2 (severe acute respiratory syndrome coronavirus 2) Omicron (B.1.1.529) variant is the antigenically most distinct variant to date. As the heavily mutated spike protein enables neutralization escape, we studied serum-neutralizing activities of naïve and vaccinated individuals after Omicron BA.1 or BA.2 sub-lineage infections in live virus neutralization tests with Omicron BA.1, Omicron BA.2, wildtype (WT, B1.1), and Delta (B.1.617.2) strains. Serum samples obtained after WT infections and three-dose mRNA vaccinations with and without prior infection were included as controls.

**Results:**

Primary BA.1 infections yielded reduced neutralizing antibody levels against WT, Delta, and Omicron BA.2, while samples from BA.2-infected individuals showed almost no cross-neutralization against the other variants. Serum neutralization of Omicron BA.1 and BA.2 variants was detectable after three-dose mRNA vaccinations, but with reduced titers. Vaccination-breakthrough infections with either Omicron BA.1 or BA.2, however, generated equal cross-neutralizing antibody levels against all SARS-CoV-2 variants tested.

**Conclusions:**

Our study demonstrates that although Omicron variants are able to enhance cross-neutralizing antibody levels in pre-immune individuals, primary infections with BA.1 or BA.2 induced mostly variant-specific neutralizing antibodies, emphasizing the differently shaped humoral immunity induced by the two Omicron variants. These data thus contribute substantially to the understanding of antibody responses induced by primary Omicron infections or multiple exposures to different SARS-CoV-2 variants and are of particular importance for developing vaccination strategies in the light of future emerging variants.

## Introduction

The SARS-CoV-2 (severe acute respiratory syndrome coronavirus) Omicron (B.1.1.529) variant of concern (VOC) is now prevalent in large parts of the world. It has been divided into several lineages [BA.1 to BA.5, their descendants and BA.1/BA.2 recombinant forms^1^ (1)], which are characterized by a heavily mutated spike protein, leading to substantial escape from antibodies induced by previous infections and/or vaccinations [reviewed in (2–5)]. The rapid increase in Omicron BA.2 infections, which has recently replaced BA.1 as the dominant variant^2^ (6), indicates that it is more transmissible than BA.1 (7) and/or may escape antibody-mediated immunity, potentially including the protection gained from Omicron BA.1 infections (8, 9).

The data reported so far yielded a heterogeneous picture with respect to the degree of cross-neutralization between Omicron and pre-Omicron variants, with most studies using samples from pre-immune, i.e. vaccinated and/or convalescent, individuals (10–17). Here, we determined neutralizing antibody titers in serum samples collected after primary as well as vaccination-breakthrough infections with Omicron variants BA.1 or BA.2. Using live virus assays with an ancestral wildtype (WT) strain and three VOCs (Delta, Omicron BA.1, Omicron BA.2), we found that infections with Omicron variants boostered cross-neutralizing antibodies in pre-immune individuals. Primary infections with one of the Omicron sub-lineages, however, induced mainly variant-specific neutralizing antibodies; particularly BA.2 infections generated a sub-lineage-specific neutralization pattern.

## Methods

### Human Serum Samples

Serum samples were collected from non-vaccinated patients hospitalized after a SARS-CoV-2 WT infection between March and November 2020 (before the emergence of VOCs) (**Table S1**). Samples from non-vaccinated individuals with primary SARS-CoV-2 Omicron infections were collected at primary health-care centers between January and April 2022, and identification of SARS-CoV-2 infection was based on PCR testing. Omicron-infected patients developed various symptoms including fever, cough, headache, rhinitis, sore throat, muscle or body aches, fatigue, diarrhea, and hoarseness. According to the family physicians, none of the patients suffered from an underlying immune deficiency, and no particular pre-existing conditions were reported. Samples from vaccinated and/or SARS-CoV-2 infected individuals were sent to the diagnostic laboratory of the Center for Virology, Medical University of Vienna, Austria. Individuals were regularly tested by SARS-CoV-2 PCR and/or lateral flow assays under the COVID-19-mass-testing program in Austria, which excluded additional SARS-CoV-2 infections.

### Cell Lines

Vero E6 cells (ECACC #85020206) were from the European Collection of Authenticated Cell Cultures (ECACC), and VeroE6-TMPRSS2 cells were kindly provided by Anna Ohradanova-Repic. Both cell lines were cultured in Dulbecco’s Modified Eagle Medium (Gibco, Thermo Fisher Scientific, Waltham, MA, USA) containing 10% Fetal Bovine Serum (Capricorn Scientific GmbH, Ebsdorfergrund, Germany) and 1% Penicillin-Streptomycin-Glutamine (Gibco, Thermo Fisher Scientific) at 37°C and 5% CO2. The cell lines were tested negative for mycoplasma contamination by the MycoAlertTM Mycoplasma Detection Kit (Lonza Group Ltd, Basel, Switzerland) in regular intervals.

### SARS-CoV-2 Isolates

SARS-CoV-2 strains were isolated from nasopharyngeal swabs from COVID-19 patients using either Vero E6 cells (WT D614G and Delta) or VeroE6-TMPRSS2 cells (both Omicron variants) (18–20). The sequences of the strains were determined by next generation sequencing and uploaded to the GISAID database (WT, B.1.1 with the D614G mutation: EPI_ISL_438123; Delta, B.1.617.2-like, sub-lineage AY.122: EPI_ISL_4172121; Omicron, B.1.1.529+BA.*, sub-lineage BA.1.17: EPI_ISL_9110894; Omicron, B.1.1.529+BA.*, sub-lineage BA.2.9: EPI_ISL_11110193. Pango lineages were determined with Pango v.4.0.6, Pango-data v1.6.) (18–20).

### Omicron BA.1 and BA.2 Variant Identification

Identification of Omicron BA.1 and BA.2 variants was performed with nasopharyngeal swabs obtained from 19 patients (n=8, primary Omicron infection; n=11, Omicron vaccination breakthrough infections) using the mutation assay VirSNiP SARS-CoV-2 Spike S371L S373P (TIB MOLBIOL, Berlin, Germany). Characteristic melting peaks for the mutations S371LS373P and S371FS373P indicated an infection with Omicron BA.1 and BA.2, respectively.

### Neutralization Assays

The live virus neutralization test (NT) was performed as described previously (18–20). Two-fold serial dilutions of heat-inactivated serum samples were incubated with 50–100 TCID50 SARS-CoV-2 for one hour at 37°C before the mixtures were added to Vero E6 cells. After three to five days at 37°C, NT titers were expressed as the reciprocal of the serum dilution required for prevention of virus-induced cytopathic effects (CPE), which was assessed by microscopy and validated by two different operators. At least one negative and two positive human polyclonal samples were included as controls in each assay. NT titers of serum samples ≥10 were considered positive.

### Statistical Analyses

Statistical analysis was performed with GraphPad Prism 9.3.1. The Mann-Whitney test was used for pairwise comparisons. The Kruskal-Wallis test followed by Dunn´s multiple comparison was used for analyzing four groups. P values < 0.05 were considered significant.

## Results

We analyzed the neutralizing capacity of serum samples obtained after primary Omicron BA.1 and BA.2 infections as well as after mRNA-vaccination-breakthrough infections with both Omicron variants against a WT strain (isolated early in the pandemic with the D614G mutation) and the three variants of concern Delta, Omicron BA.1, and Omicron BA.2 in live virus neutralization assays (see Methods). As controls, we included samples from hospitalized patients obtained after primary WT infections as well as from individuals after three doses of an mRNA vaccine with and without prior SARS-CoV-2 infection. The characteristics of these cohorts are summarized in **Table 1** and **Tables S1**, **S2**.

**Table 1:** Demographics of study cohorts and sampling time points.

	Vaccinees (3 mRNA vaccinations)	WT convalescent + 3 mRNA vaccinations	WT primary infection	Omicron BA1 primary infection	Omicron BA2 primary infection	Omicron breakthrough infections
**n**	15	9	11	22	21	43
**Median age in years [range]**	39 [23-59]	42 [27-60]	33 [19-52]	42 [4-64]	45 [24 - 81]	40 [18 - 76]
**Male sex (% of N)**	2 (13)	3 (33)	9 (82)	7 (32)	10 (48)	16 (37)
**Samples in dpo^a^/dpv^b^ [range]**	/	/	/	35 [16-79]	31 [10 - 90]	27 [10 - 72]
**1st samples in dpo^a^/dpv^b^ [range]**	26 [15-38]	24 [14-28]	18 [10-25]	/	/	/
**Follow-up samples in dpo^a^/dpv^b^ [range]**	106 [79-133]	/	215 [168-272]	/	/	/

^a^ dpo, days post onset of symptoms; ^b^ dpv, days post vaccination.

**Figure 1:**
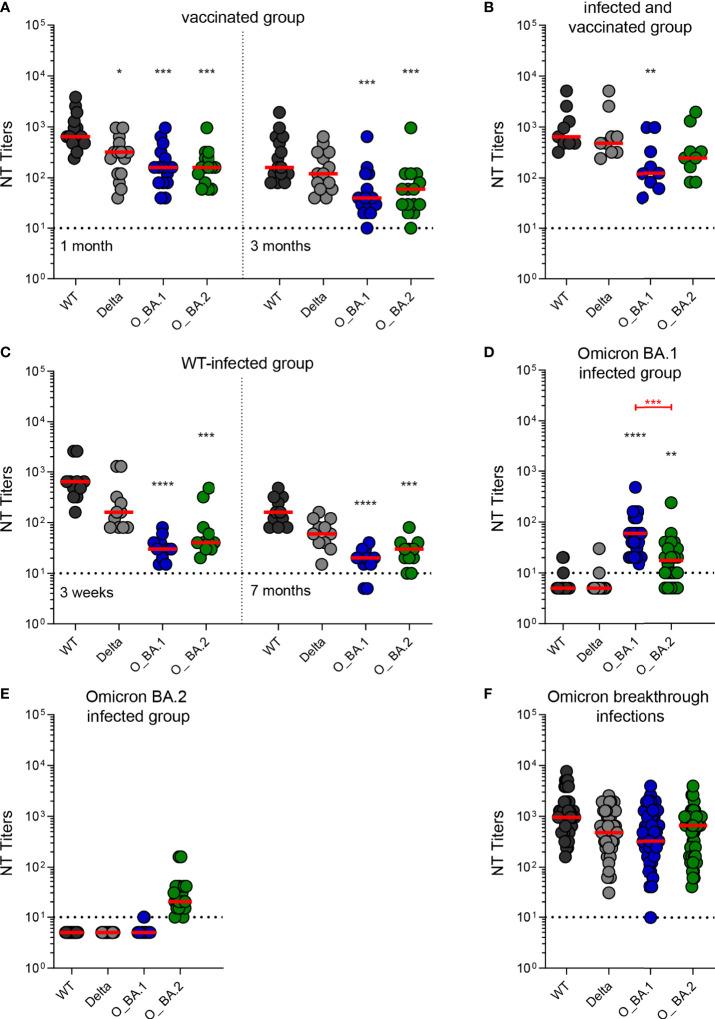
Neutralizing antibody titers against an ancestral wildtype (WT) strain and three variants of concern (Delta, Omicron BA.1, and Omicron BA.2) in post-vaccination and post-infection serum samples. **(A)** Serum samples from individuals who had received three doses of an mRNA vaccine collected one month and three months after the last vaccination (n=15). **(B)** Serum samples from individuals who were infected with a WT strain, followed by three doses of an mRNA vaccine collected three weeks after the last vaccination (n=9). **(C)** Serum samples from individuals who were infected with a WT strain collected three weeks and seven months after infection (n=11). **(D)** Serum samples obtained from individuals who had a primary infection with Omicron BA.1 collected one month after infection (n=22). **(E)** Serum samples obtained from individuals who had a primary infection with Omicron BA.2 collected one month after infection (n=21). **(F)** Serum samples from individuals who were infected with an Omicron variant and had previously been vaccinated collected one month after infection (Omicron breakthrough infections, n=43). Horizontal dotted lines show the cut-off, dots individual sera, and red lines median titers. Black asterisks indicate significant differences to WT (D614G). The Kruskal-Wallis test with Dunn’s *post-hoc* test was used for significance testing (*, p < 0.05; **, p < 0.01; ***, p <0.001; ****, p < 0.0001). Significant differences between Omicron BA.1 and BA.2 neutralization titers are indicated by red asterisks (Mann-Whitney test; ***, p < 0.001). WT, wildtype strain (B.1.1) with the D614G substitution; Delta, Delta VOC (B.1.617.2-like, sub-lineage AY.122); O_BA.1, Omicron (B.1.1.529) sub-lineage BA.1 VOC; O_BA.2, Omicron sub-lineage BA.2 VOC; NT, neutralization test.

Serum samples from vaccinees collected one and three months after the third dose of an mRNA vaccine efficiently cross-neutralized the two Omicron variants (**Figure 1A** and **Table 2**). Neutralization titers were significantly lower than for WT (**Figure 1A** and **Table 2**), but there was no significant difference between BA.1 and BA.2 neutralization (Mann-Whitney test, p > 0.05). We also detected Omicron cross-neutralization in serum samples from individuals who had a WT infection before being vaccinated three times (**Figure 1B** and **Table 2**).

**Table 2:** Serum-neutralization titers of cohorts analyzed in this study.

	n	Median [IQR]^a^ NT titer			
		WT	Delta	Omicron BA.1	Omicron BA.2
**Vaccinated group (3 doses, mRNA vaccine)**					
1-month samples^b^	15	640 (480-1120)	320 (120-400)	160 (80-280)	160 (100-240)
3-month samples^b^	15	160 (120-560)	120 (70-280)	40 (25-90)	60 (30-100)
**Convalescent and vaccinated group (WT^c^ + 3 doses mRNA vaccine)**					
3-week samples^b^	9	640 (480-1280)	480 (320-640)	120 (80-320)	240 (160-320)
**WT^c^-infected group (primary infection)**					
3-week samples^b^	11	640 (400-640)	160 (80-280)	30 (25-40)	40 (35-70)
7-month samples^b^	11	160 (100-280)	60 (40-100)	20 (15-25)	30 (20-35)
**Omicron BA.1-infected group (primary infection)**					
1-month samples^b^	22	≤10	≤10	60 (22.5-105)	17.5 (10-30)
**Omicron BA.2-infected group (primary infection)**					
1-month samples^b^	21	≤10	≤10	≤10	20 (15-40)
**Omicron-breakthrough infections**					
1-month samples^b^	43	960 (480-1920)	480 (320-1120)	320 (240-960)	640 (160-960)

^a^ IQR, interquartile range; ^b^ median values after infection or vaccination; ^c^ WT, wildtype.

Samples from individuals after primary infections with WT, Omicron BA.1, or Omicron BA.2 neutralized the heterologous strains to much lesser extents than the homologous virus (**Figures 1C–E** and **Table 2**). Neutralization titers against Omicron BA.1 and BA.2 variants were significantly reduced in samples obtained three weeks and seven months after infection with an ancestral WT strain (**Figure 1C**). Omicron BA.1-convalescents developed only very low levels of WT- and Delta VOC-neutralizing antibodies; 18/22 samples were below the cut-off (**Figure 1D**). Even BA.2 neutralization titers were significantly lower in sera of this group (**Figure 1D**). In contrast, samples from Omicron BA.2 convalescents exhibited almost no cross-neutralization of any other virus strain tested (**Figure 1E**). However, all samples from individuals after an Omicron infection who had been previously vaccinated (Omicron-breakthrough infection) were able to neutralize the Omicron BA.1 and BA.2 sub-lineages as efficiently as the WT and Delta viruses (**Figure 1F** and **Table 2**). Stratification of the Omicron-breakthrough infections according to the infecting sub-lineage revealed similar neutralization patterns of sera after Omicron BA.1 or BA.2 infection (**Figure S1**).

## Discussion

In this study, we provide a direct comparison of (cross)-neutralizing antibody responses after primary Omicron BA.1 and BA.2 infections. Consistent with the antigenic distance between Omicron BA.1 and BA.2 VOCs from pre-Omicron strains as well as from each other (8, 9, 16), we show that neutralizing antibodies present in serum samples from patients with primary Omicron infection are highly variant specific. While samples from individuals after primary BA.1 infection exhibited some cross-neutralization of BA.2 (**Figure 1D**), as reported by others (17, 21), samples from primary BA.2-infected individuals showed almost no cross-neutralizing activities against any other variant tested (**Figure 1E**). Similar results were obtained in neutralization assays performed with Omicron BA.1 and BA.2 post-infection serum samples from hamsters, with the highest levels of neutralizing antibodies against the homologous strain (22). In agreement with our results, BA.1 post-infection samples yielded stronger cross-neutralization of BA.2 than did BA.2 post-infection sera with BA.1.

**Figure 2:**
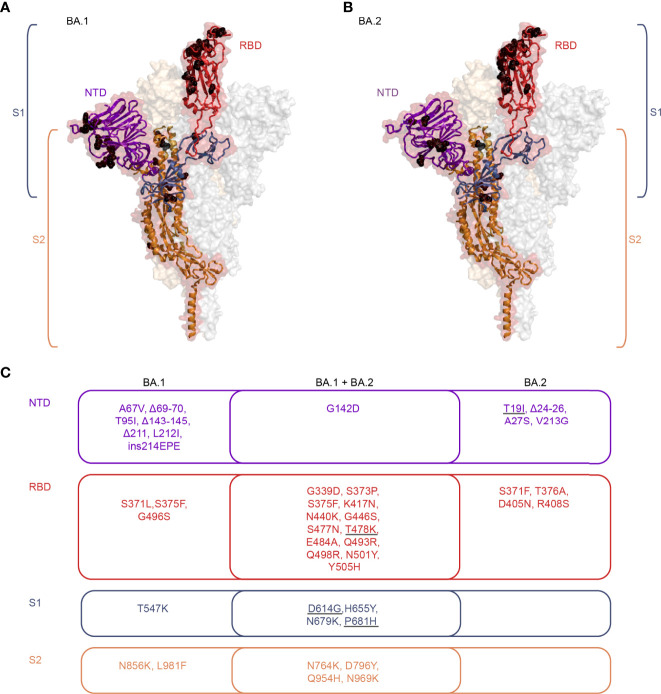
Structural representation of mutations present in Omicron BA.1 and BA.2 sub-lineages. **(A, B)** Cartoon representation of one monomer of the spike protein (side view) combined with a semi-transparent surface representation of the trimeric spike in its RBD-up-conformation [PDB 7KRR, (25)]. The three protomers are colored in red, grey, and bright orange. The S1 subunit is colored blue in the single protomer, with the RBD highlighted in red and the NTD highlighted in purple. S2 is colored in orange. The black spheres indicate the mutations present in sub-lineages BA.1 **(A)** and BA.2 **(B)**. The structures were generated with PyMol (https://pymol.org). **(C)** Venn diagram illustrating the BA.1 and BA.2 sub-lineage-specific and shared mutations, colored according to the regions highlighted in panels A and B. The grey underlines indicate mutations present in the Delta variant. RBD, Receptor-binding domain; NTD, N-terminal domain; S1, Subunit 1; S2, Subunit 2.

The mutations in the Omicron spike proteins strongly affect their antigenicity in comparison to pre-Omicron variants (23, 24), as indicated by a substantial resistance to neutralizing antibodies induced by prior SARS-CoV-2 infections and/or vaccinations (**Figures 1A–C**) [reviewed in (2–5)]. The considerable antigenic differences between the Omicron BA.1 and BA.2 spikes in the receptor-binding domain (RBD) and the N-terminal domain (23, 24) (as illustrated in **Figure 2**), the major targets of neutralizing antibodies, offer an explanation for the sub-lineage-specific neutralization profiles observed after primary BA.1 or BA.2 infections (**Figures 1D, E**). Consistent with our data, primary Omicron infections elicited RBD-specific B cells with only narrow specificity for these variants (14).

Multiple exposures to pre-Omicron-SARS-CoV-2, *via* three mRNA vaccinations (**Figure 1A**) or mixed infection-vaccination scenarios (**Figure 1B**), led to high titers with efficient neutralization of both Omicron variants. These data are in agreement with other studies that suggested an increase in the magnitude as well as the breadth of neutralizing antibody responses by repeated exposure to the original antigen (26–35). Previous data (36) suggest that a three-dose vaccination regimen enhances the breadth of antibody responses in that memory B cells are also formed against conserved epitopes. These are typically of lower immunogenicity than the more strain-specific and immunodominant epitopes, which is why repeated exposures may be needed for broader variant neutralization.

The Omicron NT titers, however, were lower in these cohorts than against pre-Omicron variants (**Figures 1A, B**) and a fast waning of neutralizing antibodies was observed (**Figure 1A**). In contrast, vaccination-breakthrough infections with Omicron induced similarly high levels of neutralizing antibodies against all variants tested (**Figure 1F**) (9, 13, 14, 37), thus leading to a strong expansion of neutralization breadth. Apparently, an adequate number of conserved epitopes for neutralizing antibodies are present in the spike proteins of Omicron and pre-Omicron variants, suggesting that Omicron-adapted vaccines might increase the effectiveness of booster immunizations.

A limitation of our study is the relatively small sample size. However, due to increasing SARS-CoV-2 immunity in the human population, it is difficult to find unvaccinated persons who have only been exposed to a single Omicron variant, which is why previous studies were performed with hamster post-Omicron infection samples (8, 22, 38). In addition, we focused on the neutralizing activities of antibodies, but also interactions with Fc receptors might be involved in protection independent of the neutralizing potency of antibodies (39).

In summary, the highly variant-specific neutralization profiles obtained with the two Omicron sub-lineages BA.1 and BA.2 in naïve individuals underscore the antigenic distance between the two variants. The rapid emergence of further immune-escape variants (Omicron BA.4 and BA.5), which have been associated with a resurgence of SARS-CoV-2 infections in South Africa (40, 41), highlights the importance of global variant surveillance and antigenic characterization to estimate the impact of the continuing SARS-CoV-2 evolution on the ongoing pandemic (42).

## Data Availability Statement

The original contributions presented in the study are included in the article/**Supplementary Material**. Further inquiries can be directed to the corresponding author.

## Ethics Statement

The analyses were approved by the Ethics Committee of the Medical University of Vienna (EK 1291/2021, EK 1926/2020, EK 2156/2019, EK 1035/2016, EK 1513/2016). Hospitalized patients provided written informed consent to participate in this study. Written informed consent was not required for the analysis of anonymized leftover samples from routine laboratory diagnosis in accordance with the national legislation and the institutional requirements.

## Author Contributions

Conceptualization, KS and JA. Investigation, KS, JA, IM, JC, and SA. Formal analysis, KS, JA, and IM. Resources, DS, MG, EH, MT, JD, OL, CB, EP-S, LW, and AZ. Writing - original draft, KS, JA, and IM. Writing - review & editing, all authors. Visualization, IM. Supervision, KS. Funding acquisition, JA and IM. All authors contributed to the article and approved the submitted version.

## Funding

IM acknowledges funding by the “Hochschuljubiläumsfonds der Stadt Wien” (Project H-334533/2021) and JA by the Medical-scientific fund of the Mayor of the federal capital Vienna [Grant Covid003].

## Conflict of Interest

The authors declare that the research was conducted in the absence of any commercial or financial relationships that could be construed as a potential conflict of interest.

## Publisher’s Note

All claims expressed in this article are solely those of the authors and do not necessarily represent those of their affiliated organizations, or those of the publisher, the editors and the reviewers. Any product that may be evaluated in this article, or claim that may be made by its manufacturer, is not guaranteed or endorsed by the publisher.

## Footnotes

1
https://www.who.int/en/activities/tracking-SARS-CoV-2-variants

2
https://www.who.int/publications/m/item/weekly-epidemiological-update-on-covid-19---22-march-2022.
