# Supplementary material for: Different Neutralization Profiles After Primary SARS-CoV-2 Omicron BA.1 and BA.2 Infections
Source: Front Immunol. 2022 Jul 19;13:946318. doi: 10.3389/fimmu.2022.946318 (PMC9344875; doi:10.3389/fimmu.2022.946318)
Supplement: Supplementary file 1 [file DataSheet_1.zip › Medits_Supplement_Revision.pdf]

Supplementary Material

Different neutralization profiles after primary SARS-CoV-2 Omicron BA.1 and BA.2 infections

Iris Medits,[1] David N. Springer,[1] Marianne Graninger,[1] Jeremy V. Camp, [1], Eva Höltl,[3] Stephan W. Aberle,[1] Marianna T. Traugott,[2] Wolfgang Hoepler,[2] Josef Deutsch,[4] Oliver Lammel,[5] Christian Borsodi,[1] Elisabeth Puchhammer-Stöckl,[1] Alexander Zoufaly,[2] Lukas Weseslindtner,[1] Judith H. Aberle,[1,#] and Karin Stiasny[1,#,*]

[1]Center for Virology, Medical University of Vienna, Vienna, Austria. [2]Department of Medicine IV, Clinic Favoriten, Vienna Healthcare Group, Vienna, Austria. [3]Center for Public Health, Medical University of Vienna, Vienna, Austria. [4]Practice Dr. Deutsch, Völkermarkt, Austria. [5]Practice Dr. Lammel, Ramsau am Dachstein, Austria.

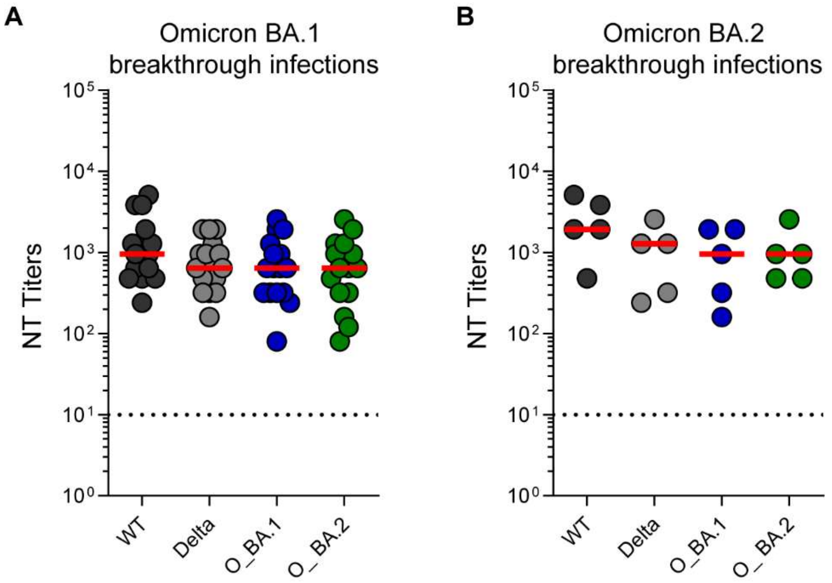

Figure S1: Neutralizing antibody titers against an ancestral wildtype (WT) strain and three variants of concern (Delta, Omicron BA.1, and Omicron BA.2) in serum samples from vaccinated individuals who were infected with Omicron (breakthrough infections)
(A) Serum samples from previously vaccinated individuals who were infected with Omicron BA.1 (Omicron BA.1-breakthrough infections, n=16).
(B) Serum samples from previously vaccinated individuals who were infected with Omicron BA.2 (Omicron BA.2-breakthrough infections, n=5).

Horizontal dotted lines show the cut-off, dots individual sera, and red lines median titers.

Table S1. Demographic data of the WT infected cohort

N	11
Demographic characteristics	
Median age in years [range]	33 [19-52]
Sex (male/female)	9m/2f
Time from symptom onset to serum sample collection [days]	35 [16-79]
Hospitalized	11
ICU	7
Comorbidities	10
Obesity	10
Diabetes mellitus type 2	2
Cardiovascular disease	1
Multiple sclerosis	1
Psoriasis	1
St.p. HTX	1
St.p. thyroidectomy	1
Ventilation form	
Invasive mechanical ventilation	5
Non-invasive ventilation	3
High-flow nasal cannula oxygen therapy	2

St.p., status post; HTX, heart transplantation; ICU, intensive care unit

Table S2. Characteristics of vaccinated patients with Omicron breakthrough infections

Sample	Age	Sex	dpo (serum sample)	Infection prior to vaccination	Vaccination			Omicron infection	
					1st vaccination	2nd vaccination	3rd vaccination	dpv	Sub-lineage
1	26	m	21	N	Moderna	Moderna	Moderna	27	BA.1[a]
2	22	m	21	N	Pfizer	Pfizer	Pfizer	34	BA.1[a]
3	25	f	21	N	Moderna	Moderna	N	212	BA.1[a]
4	43	m	13	N	Pfizer	Pfizer	N	123	BA.1[a]
5	39	f	15	N	Pfizer	Pfizer	Moderna	144	BA.1[a]
6	37	f	23	N	Pfizer	Pfizer	Pfizer	44	BA.1[a]
7	32	f	18	N	Pfizer	Pfizer	Moderna	141	BA.1[a]
8	29	m	22	Y	Astra	Astra	Pfizer	34	BA.1[a]
9	20	m	20	Y	Pfizer	Pfizer	N	104	BA.2[b]
10	22	m	16	Y	Johnson	Pfizer	N	61	BA.1[a]
11	25	f	32	N	Pfizer	Pfizer	N	84	BA.1[a]
12	21	f	10	N	Pfizer	Pfizer	Moderna	164	BA.1[b]
13	76	f	30	N	Pfizer	Pfizer	Pfizer	110	BA.1[b]
14	33	f	36	N	Pfizer	Pfizer	Pfizer	105	BA.1[b]
15	47	f	20	N	Pfizer	Pfizer	Pfizer	139	BA.1[b]
16	28	f	22	N	Pfizer	Pfizer	Moderna	110	BA.2[b]
17	51	f	27	N	Pfizer	Pfizer	Pfizer	114	n.a
18	53	f	20	N	Pfizer	Pfizer	Pfizer	101	BA.1[b]
19	55	m	17	N	Pfizer	Pfizer	Pfizer	106	BA.1[b]
20	57	f	27	N	Pfizer	Pfizer	Pfizer	190	BA.2[b]
21	27	m	28	N	Pfizer	Pfizer	Moderna	59	BA.2[b]
22	62	m	n.a.	N	Pfizer	Pfizer	Pfizer	n.a.	n.a.
23	n.a.	f	n.a.	N	Pfizer	Pfizer	Pfizer	n.a.	n.a.
24	45	f	32	N	Pfizer	Pfizer	Pfizer	93	n.a.
25	25	m	35	N	Astra	Astra	Moderna	52	n.a.
26	23	f	35	N	Pfizer	Pfizer	Pfizer	81	n.a.
27	64	m	31	N	Pfizer	Pfizer	Pfizer	76	n.a.
28	40	m	30	N	Pfizer	Pfizer	Pfizer	112	n.a.
29	33	m	54	N	Pfizer	Pfizer	Pfizer	99	n.a.
30	29	m	55	N	Pfizer	Pfizer	N	85	n.a.
31	62	f	n.a.	N	n.a.	n.a.	n.a.	n.a.	n.a.
32	52	f	n.a.	N	n.a.	n.a.	n.a.	n.a.	n.a.
33	51	f	n.a.	N	n.a.	n.a.	n.a.	n.a.	n.a.

34	27	f	n.a.	N	n.a.	n.a.	n.a.	n.a.	n.a.
35	54	f	n.a.	N	n.a.	n.a.	n.a.	n.a.	n.a.
36	18	f	72	N	n.a.	n.a.	n.a.	62	n.a.
37	57	f	n.a.	N	n.a.	n.a.	n.a.	n.a.	n.a.
38	70	f	44	N	n.a.	n.a.	n.a.	120	n.a.
39	40	f	44	N	n.a.	n.a.	n.a.	120	n.a.
40	49	f	26	N	Pfizer	Pfizer	N	127	n.a.
41	49	m	26	N	Pfizer	Pfizer	Pfizer	127	n.a.
42	52	m	n.a.	Y	n.a.	n.a.	n.a.	n.a.	n.a.
43	34	f	30	Y	Pfizer	Pfizer	N	109	BA.2[b]

dpo, days post onset of symptoms; dpv, days post vaccination; n.a., data not available; N, no; Y, yes.

[a] Omicron infection during BA.1 wave in Austria (January 2022 – February 2022).
[b] Omicron BA.1 or BA.2 infection was confirmed using the mutation assay VirSNiP SARS-CoV-2 Spike S371L S373P, as described in Methods.
